# A Systematic Review of Communication for Behavioural Impact (COMBI) as a Dengue Prevention Programme

**DOI:** 10.21315/mjms-07-2024-527

**Published:** 2025-06-30

**Authors:** Priya Dharishini Kunasagran, Syed Sharizman Syed Abdul Rahim, Gary Goh Chun Chao, Adora J Muyou, Zulkhairul Naim Sidek, Aizuddin Hidrus, Azman Atil, Rahmat Dapari

**Affiliations:** 1 Department of Public Health Medicine, Faculty of Medicine and Health Sciences, Universiti Malaysia Sabah, Kota Kinabalu, Sabah, Malaysia; 2 Borneo Medical and Health Research Centre, Faculty of Medicine and Health Sciences, Universiti Malaysia Sabah, Kota Kinabalu, Sabah, Malaysia; 3 Department of Community Health, Universiti Putra Malaysia, Serdang, Selangor, Malaysia

**Keywords:** dengue, COMBI, barriers, facilitating factors, effectiveness

## Abstract

The World Health Organization’s Communication for Behavioural Impact (COMBI) strategy aims to promote healthy behaviours through integrated communication. Malaysia has adopted the COMBI approach to combat dengue; however, sustaining these efforts presents ongoing challenges. This review explored the facilitating factors and barriers associated with challenges in the sustainability of Malaysia’s COMBI programme for dengue prevention. We used the SPIDER framework (Sample, Phenomenon of Interest, Design, Evaluation, Research Type) to identify relevant articles, and we searched Scopus, PubMed, ScienceDirect, BMJ, Sage, and Google Scholar. Our search followed PRISMA guidelines, using Boolean operators “AND” and “OR” to connect keywords like “dengue” and “Communication for Behavioural Impact (COMBI).” The review included five articles, which were appraised by the Joanna Briggs Institute (JBI) Critical Appraisal Tool. Findings indicate that COMBI programmes significantly enhance knowledge, attitudes, and practices (KAP) and reduce both dengue cases and mosquito breeding sites. The reviewed articles identified nine themes as either facilitating factors or barriers to the sustainability of COMBI: community engagement, leadership, political stability, mass media, public health campaigns, funding, human resources, multi-sectoral collaboration, and migration of residents. Identifying factors and barriers is key to improving the effectiveness and sustainability of COMBI programmes. This review highlights the importance of mixed methods research and community-centric engagement in addressing community issues and refining COMBI strategies. These comprehensive approaches are crucial for sustaining community involvement and ensuring the effectiveness of dengue prevention, thereby contributing to the long-term success of public health interventions in Malaysia and globally.

## Introduction

The World Health Organization’s Communication for Behavioural Impact (COMBI) strategy, a significant development by the World Health Organization, integrates social and behavioural communication into public health programmes. It plays a crucial role in influencing community behaviour and encouraging healthy practices, thereby increasing the efficiency of dengue preventive efforts (1). This technique is based on the concept that disseminating information alone is insufficient to achieve long-term changes in health; instead, it is crucial to actively engage with communities and influence their behaviours (1). This approach has been instrumental in public health victories, such as eradicating leprosy in India and Mozambique, eliminating lymphatic filariasis in India and Zanzibar, and preventing dengue fever in Malaysia. These successes underscore the potential of the COMBI strategy in combating various diseases through improved communication and social mobilisation, offering hope for its effectiveness in Malaysia’s dengue prevention efforts (2).

Central to COMBI’s effectiveness in dengue prevention are five fundamental principles: i) public relations, advocacy, and administrative mobilisation, which involve coordination and advocacy meetings with health offices and COMBI teams; ii) community mobilisation, which engages the community in participatory activities; iii) advertising to spread health information; iv) interpersonal communication for direct outreach during outbreaks; and v) point-of-service promotion for delivering targeted health messages (3). These principles aim to foster community involvement and behavioural change, which are crucial for the sustainability of health interventions. The recognition that dengue transmission is influenced by both environmental factors and human behaviours—individually and collectively—has motivated the implementation of the COMBI programmes in Malaysia to address the complexity of dengue transmission. Research conducted in several states in Malaysia has shown that COMBI can dramatically increase community knowledge, attitudes, and practices (KAP) related to dengue prevention (4–6). The findings of this study indicate that combining tailored communication strategies with community involvement can significantly improve dengue control outcomes.

Community-led initiatives and active engagement in programmes like those in Pakistan underscore the crucial role of local leadership in the success of health-related projects (6, 7). Individual perceptions, access to information, and behaviours related to dengue play a critical role in disease management, underscoring the importance of health beliefs in shaping community responses to prevention efforts (8). However, obstacles like limited cooperation between health officials and local communities, as well as the community’s dependence on health authorities to initiate action, present significant barriers to the sustained effectiveness of these interventions (5, 9, 10). Therefore, this review explored the knowledge, facilitating factors, and barriers associated with Malaysia’s COMBI programme by closely reviewing literature related to COMBI in dengue prevention. The review emphasised study designs and methods, topics covered, and the determinants that either facilitated or hindered the long-term sustainability of these interventions. Sustained community involvement and effective dengue prevention are crucial in ensuring the long-term success of public health interventions in Malaysia and globally.

## Methods

The study followed a methodology that involved a comprehensive evaluation of critical findings from various studies related to COMBI as a dengue prevention programme. The author adhered to the Preferred Reporting Items for Systematic Reviews and Meta-Analysis (PRISMA) guidelines when assessing and selecting research articles.

### Search Strategy

The mnemonic SPIDER, which stands for Sample, Phenomenon of Interest, Design, Evaluation, and Research type, was used. Here, “Sample” refers to the community engaged in COMBI, considered the primary group of interest, “Phenomenon of Interest” is the activities related to dengue prevention within the community, and “Design” encompasses methods such as questionnaires, surveys, focus groups, and population studies chosen for the research. “Evaluation” pertains to reviewing the impact on sustainability, effectiveness, and behaviour regarding dengue prevention, and “Research type” includes qualitative, quantitative, and mixed methods research approaches. These elements shaped the strategy for searching and selecting pertinent research articles. The search strategies were developed and fine-tuned by the study’s reviewers, who likely used relevant keywords and tailored them to the syntax required by each database. The search results were then organised and managed systematically by consolidating them into an Excel spreadsheet.

Scopus, PubMed, ScienceDirect, BMJ, and Google Scholar databases were last searched on 23 October 2023 to identify potentially relevant articles in adherence to the PRISMA guidelines. The literature search strategy incorporated the use of common Boolean operators such as “AND” and “OR,” as well as their counterparts in Medical Subject Heading (MeSH) terminology, to link keywords relevant to the research topic and title. To search across five databases, the keywords “dengue” AND “Communication for Behavioural Impact” OR “COMBI” were entered into the search fields, with additional filters applied to refine the results based on the specific criteria.

### Inclusion and Exclusion Criteria

Articles were included if they were: i) focused on COMBI related to dengue prevention activities; ii) published between 2013 and 2023; iii) written in the English language; iv) had a full-text version of the studies available; and v) used qualitative, quantitative, or mixed methods. Articles were omitted from consideration if they met any of the following criteria: i) published before the year 2003; ii) in languages other than English, as the authors decided against using translation software due to concerns about missing subtle language details; iii) merely study protocols without complete results; iv) pre-prints or had not been formally published; v) categorised as short communications, letters, editorials, or commentaries; and vi) systematic reviews. The selection criteria ensured that the chosen articles aligned with the specific aims of the study, emphasising relevance, recency, and availability in the English language. This process aimed to preserve the focus and credibility of the systematic review by omitting specific document types, such as other systematic reviews and brief communications.

### Search Outcome

**Figure 1:**
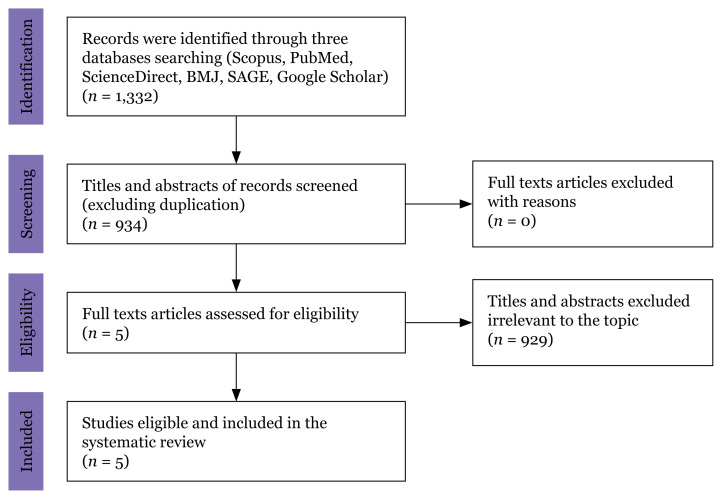
PRISMA flow diagram for the study selection

The selection of articles for the study followed a four-step PRISMA diagram process outlined in Figure 1 and organised in a spreadsheet. Initially, the team consolidated articles from all database searches during the identification phase, formulating a research question and developing a thorough search strategy to find pertinent articles. During the screening phase, team members independently reviewed the titles and abstracts to determine initial article relevance based on the inclusion criteria. Disagreements were resolved through discussion or by consulting a third reviewer. During the eligibility phase, the team assessed full-text articles to ensure they met the inclusion criteria, excluding those that did not and documenting the reasons for their exclusion. The articles that passed this stage underwent a methodological quality assessment. In the final step, a systematic review was conducted on the articles relevant to the study’s focus. Search results were exported to a bibliographic programme like Mendeley to facilitate data management and initial review. Subsequently, selected articles underwent critical appraisal using suitable tools. Other researchers re-evaluated the articles from identification to eligibility as part of the selection process. Following this, data from the qualifying studies were independently extracted according to the systematic review criteria, preparing for the analysis of the findings.

### Quality Assessment

The researcher independently evaluated the quality of all five studies using the Critical Appraisal Guidelines provided by the Joanna Briggs Institute (JBI). Any differences in assessment were resolved through discussion with other researchers until a consensus was reached. The methodological quality of the articles in this systematic review was determined using the JBI Critical Appraisal Tool, which examines the essential aspects of a research study and helps appropriately assess the contribution of each study to the overall synthesis of findings. This tool scrutinises the research methodology and assesses how well the study has addressed potential biases in its design, implementation, and analysis. Studies that demonstrated high methodological quality were given more significant consideration, ensuring the review reflected a reliable and comprehensive overview of the evidence. The JBI tool is adapted to the specific type of research being reviewed, including qualitative studies which use a specialised checklist. This enables a thorough evaluation of critical factors such as the suitability of the research design, the rigour in data collection, the transparency of the analysis, the reflexivity of the researchers, and the clarity of the reported results. For the quality assessment, we applied ten criteria from the checklist, scoring each as yes, no, unclear, or not applicable. Criteria marked as “yes” were awarded 1 point, while the rest received 0 points. The total scores were then calculated and expressed as a percentage.

For qualitative research, we employed the JBI Checklist for Qualitative Studies to carefully examine each study for straightforward research questions or objectives, the appropriateness of the design, the effectiveness of the sampling strategy, the thoroughness of data collection methods, the reliability of statistical analyses, and the acknowledgement of potential biases. This approach allowed the identification of studies with both methodological strengths and weaknesses, contributing to a more nuanced understanding of the evidence base. The quality assessment for these articles also involved eight specific checklist criteria with a similar scoring system. This meticulous evaluation process using the JBI Critical Appraisal Tool enhances the credibility of our systematic review and solidifies the reliability of the conclusions drawn from the aggregated evidence.

## Results

The initial search found 1,332 articles. After eliminating duplicate entries, we identified the titles and abstracts of the remaining records, which were then screened for relevance to the topic through the searches before the duplicate records were removed. As a result, 929 articles were excluded due to a lack of relevance (Figure 1). After completing the screening process, we assessed the remaining five articles for eligibility. These five papers were included in the systematic review as they met all the eligibility criteria. This review encompasses five studies published between 2003 and 2023, all of which examine the COMBI programme for dengue prevention. Three of these studies employed quantitative methods, one incorporated the qualitative method, and the fifth used mixed methods. The research was conducted exclusively in different regions of Malaysia, focusing on communities and health workers as the study populations. Tables 1 and 2 summarise the key attributes of the five studies.

Our systematic review utilised thematic analysis to evaluate both the facilitating factors and barriers that influence the long-term sustainability of COMBI programmes in preventing dengue. These themes provide insights into the effectiveness of public health efforts, particularly in dengue prevention through the COMBI programme. Table 3 provides an overview of the themes identified across the five studies. Based on the reviewed articles on COMBI, we developed nine themes: community engagement or commitment, leadership, political stability, mass media, public health campaigns, funding, human resources (health office), multi-sectoral collaboration, lack of training and migration of residents. The themes are categorised as either barriers (B), facilitating factors (FF), or elements that are not available (N/A) to the studies reviewed.

Based on the research carried out between 2013 and 2016, COMBI programmes have significantly influenced community KAP associated with dengue prevention, as well as the number of dengue cases and mosquito breeding sites (11, 13, 16). KAP were reported as facilitating factors in three studies, signifying that improved community understanding and behaviours are pivotal in reducing dengue incidence. These studies also used entomological findings as evidence to analyse reductions in dengue cases and correlate them with improvements in KAP regarding dengue prevention in the study area (11, 13, 16). Two studies also highlighted community engagement as a facilitator, underscoring the importance of active community participation in the success of these interventions, as evidenced in the studies carried out in Hulu Langat and Nilai (13, 14). However, these factors were also identified as barriers in two other studies. In addition, good leadership emerged as a key facilitating factor in one study, highlighting the role of strong leadership in steering COMBI programmes toward their objectives. Using qualitative research techniques such as in-depth interviews and focus group discussions, the study explored the characteristics of communities actively participating in COMBI programmes compared to those experiencing a decline in their level of activity involvement (15). To ensure the success and long-term viability of COMBI activities, the results highlighted the significance of leadership, community participation, and multi-sectoral cooperation as critical components.

Political stability was marked as a barrier, suggesting that a stable political climate is necessary for the smooth operation of public health programmes (11, 15). Two studies identified funding challenges, indicating that financial constraints may limit the reach and sustainability of the COMBI programmes (11, 15). Human resource limitations within health offices were also seen as a barrier, highlighting the need for adequate staffing to implement these programmes effectively. Additionally, a lack of training was mentioned as a barrier, implying that proper education and workforce preparation are essential for the success of dengue prevention strategies. In one study, mass media campaigns were a practical tool for raising public awareness and encouraging behaviour change to help prevent dengue. Furthermore, it advocates for ongoing innovation, execution, and evaluation of similar programmes to effectively combat dengue fever, particularly in regions where the disease is endemic (11). Finally, one study identified resident migration as a barrier, highlighting the difficulty of maintaining consistent community-based interventions amid population movement (13). Addressing these barriers while capitalising on facilitating factors is crucial to enhancing the effectiveness and sustainability of COMBI programmes in the fight against dengue.

## Discussion

In this systematic review, we examined five articles related to the COMBI programmes in Malaysia. The themes identified are crucial elements influenced by both community engagement and external factors that contribute to the sustainability of the COMBI programmes. This review identifies that KAP, community engagement, and strong leadership as pivotal factors for the continuity of the COMBI programme within the community. Conversely, external factors such as political instability, insufficient training, limited human resources, inadequate multi-sectoral collaboration, lack of financial support, and limited mass media presence play a substantial role in either bolstering or undermining the long-term sustainability of the COMBI programme.

The effectiveness of COMBI programmes relies heavily on the interplay between KAP, community engagement, and leadership. The KAP aspect was consistently highlighted as a facilitating factor, illustrating that enhanced understanding and behavioural change within the community is vital in preventing dengue. Studies have demonstrated that when individuals are well-informed about dengue prevention and control measures, their behaviours align more closely with public health recommendations, thereby reducing the incidence of the disease (5, 17, 18). On the other hand, Burkina Faso, a West African country, reported that communities with adequate knowledge and awareness about dengue and malaria prevention were less associated with disease infection as the community implemented precautionary measures (19). Studies indicate that sustaining elevated KAP levels is essential since community KAP can decline without regular training or health education, necessitating ongoing commitment and resource allocation (20, 21).

Community engagement is another critical factor, with evidence suggesting that the active involvement of community members is associated with better outcomes in health interventions. Such engagement can take many forms, from participation in public health campaigns to community-driven initiatives for controlling mosquito breeding. Effective community initiatives rely on local support, which can be achieved through targeted education and mobilisation efforts that align with community values and address local issues (22). Although localised support is essential for community-based interventions, education and mobilisation may not always be sufficient. It is important to recognise that communities have changing demands and challenges. Therefore, flexibility, adaptability, and broad-based relationships beyond the local setting may significantly enhance the effectiveness and sustainability of these initiatives (23). Engaging with government, non-profit, and commercial collaborators provides valuable resources, expertise, and perspectives for addressing challenging circumstances (21). This broader engagement can also expand and replicate successful programmes, increasing their impact on society.

Strong leadership within the COMBI programmes were identified as a facilitator, indicating that solid and effective leadership is vital for programme success. Leaders play a pivotal role in organising efforts, galvanising community action, and maintaining the momentum of the programmes. They are often the linchpins who translate policy into action and oversee the operational aspects of the interventions. Effective leadership and commitment from the community leaders were identified as critical drivers in a successful community-based intervention to prevent disease (24). Moreover, studies found that continuous funding by the authorities for the implementation of the programme and recognition of the efforts taken by the community leaders to assist in disease prevention and control in their respective localities has contributed to the success of community-based activities (8, 25–27). On the other hand, political stability emerges as a vital external factor influencing the efficacy of COMBI programmes. When political conditions are stable, public health initiatives are more likely to receive continuous support for sustained operation and success. Conversely, political instability can disrupt funding, policy continuity, and resource allocation, which are crucial for maintaining and scaling health programmes (28). Studies have demonstrated that political structures at higher levels of governance influence how individuals and organisations implement dengue prevention and control measures at lower levels of the healthcare system (29).

Mass media is a powerful tool for public health communication, offering a broad platform for extensive education campaigns that can reach wide audiences. The reviewed studies highlight the efficacy of mass media campaigns in enhancing awareness about dengue prevention, leading to behavioural change and mobilising communities (11, 30). Similarly, in Paraguay, a community-based intervention leveraging information and communication technologies alongside robust communication strategies proved effective for dengue prevention (31). Comparable outcomes were seen in Surabaya, where forming a community social group in cooperation with health authorities supported community-wide efforts to prevent dengue (32). Funding is a persistent barrier identified in the systematic review. Adequate financing is required for the initial rollout of COMBI programmes and their ongoing operations and expansion. Insufficient funding can limit the scope of activities, reduce the availability of resources, and hamper the ability to respond to emerging health challenges (21, 23, 33). Human resources, particularly in health offices, were highlighted as both a barrier and a critical need. The effectiveness of any public health programme is directly tied to the availability of trained personnel who can implement strategies, educate the community, and provide essential services. Shortages or limitations in this area can severely restrict programme reach and effectiveness, prioritising health workers’ recruitment, training, and retention (15, 29, 33).

This systematic literature review provides significant insights into public health interventions, which focus on the effectiveness of the Communication for Behavioural Impact (COMBI) programme in preventing dengue fever in Malaysia. COMBI programmes can influence community involvement in dengue prevention efforts by enhancing knowledge and behavioural habits, ultimately reducing dengue cases and mosquito breeding sites. However, understanding the human behavioural component—including the motivations, experiences, and challenges of the volunteers driving these initiatives—could offer deeper insights into the factors that contribute to or hinder the success of these programmes. While the quantitative measures of epidemiology, entomology, and KAP provide invaluable data on the outcomes of the COMBI programmes, exploring individual behavioural factors can offer a better understanding of these factors. This understanding is essential for developing health communication techniques that are more individualised, culturally sensitive, and sustainable and that resonate with both the volunteers and the larger community.

### Limitations and Suggestions

The limited number of articles on COMBI is likely due to the specific keywords used, which were chosen to deepen the understanding of COMBI. Additionally, it is essential for future research to include information from different geographical areas to provide a complete overview of the factors that facilitate or hinder the sustainability of the COMBI programme as a dengue prevention initiative.

## Conclusions

In conclusion, while the COMBI programme in Malaysia shows promise in preventing dengue, it is crucial to conduct a complete assessment that considers both quantitative results and qualitative experiences to maximise the effectiveness of these interventions, particularly among the volunteers involved in the programme. These can improve the long-term sustainability and effectiveness of COMBI programmes, ensuring that these programmes continue to be an essential component of dengue preventive initiatives in Malaysia and other comparable settings globally.

## Figures and Tables

**Table 1:** Overview of articles based on authors, title, country, and source

Authors	Title	Country	Source
Ismail et al. (11)	COMBI program in dengue prevention evaluation: mixed methods approach	Malaysia	Scopus
Suhaili et al. (12)	Applying COMBI in the prevention and control of dengue in Johor Bahru, Johore, Malaysia	Malaysia	Scopus
Rozhan et al. (13)	The COMBI program in the prevention and control of dengue – the Hulu Langat experience	Malaysia	Google Scholar
Hod et al. (14)	The COMBI approach in managing dengue cases in an urban residential area, Nilai, Malaysia	Malaysia	PubMed
Suraiya et al. (15)	COMBI approach as community-based intervention in dengue control through leadership	Malaysia	Scopus

COMBI = communication for behavioural impact

**Table 2:** Overview of article methodology and outcome

Objective	Study population	Study design	Methodology	Type of instrument	Outcome measures	Studies outcome
Assessing COMBI Programme impact in Selangor’s Hulu Langat	Community and health officer	Mix-method	Focus group discussion, self-administrated questionnaire, secondary data collection (epidemiology and entomological)	KAP, semi-structured questionnaires (about the COMBI programme and problems encountered)	Knowledge, attitude, practice, reduction in the number of dengue cases Impact of COMBI	Knowledge was equal in both the target and control groups, while attitude and practice were slightly higher in the target group Gender, age, occupation, and income level were not found to be significantly influenced by KAP
Provides highlights on COMBI intervention	Community	Cross-sectional	Self-administered questionnaire	Pre- and post-COMBI KAP surveys and treatment-seeking surveys	Knowledge, attitude, practice, and treatment-seeking behaviour	Both the KAP and treatment-seeking behaviour improved compared to the pre-survey
Discussed the rollout of the COMBI programme through market analysis	Community	Cross-sectional	Entomological survey data collection	N/A	Entomological Surveys (Aedex Index, Breteau Index, Container Index, Breeding Containers) and reduction in the number of dengue cases	Evidence of reduction in the number of dengue cases Reduction in breeding places
Evaluating COMBI strategy’s impact on dengue control in Nilai’s Taman Desa Kolej	Community	Cross-sectional	Self-administered questionnaire	Pre- and post-COMBI KAP survey	Knowledge, attitude, practice and Entomological Surveys (Bare Leg Catching (BLC), CDC Light Trap, Ovitrap index)	KAP improved from pre- to post-intervention Evidence of cases correspondent with entomological findings
Analysing the attributes of communities that actively implement COMBI strategies compared to those with decreased engagement	Community and health officer	Qualitative	In-depth interview, focus group discussion	Semi-structured surveys are conducted in knowledge, training, COMBI structure, leadership, relationships among members and coordinators, and community	Sustainability of COMBI	Strong leadership that fully grasps the concept of community mobilisation is more capable and confident in conducting COMBI, and their success is evident

COMBI = communication for behavioural impact; KAP = knowledge, attitudes, and practices; N/A = not available

**Table 3:** Overview of facilitating factors and barriers in the sustainability of COMBI

Authors	KAP	Community engagement	Leadership	Political stability	Mass media public health campaign	Funding	Human resource (health office)	Multi-sectoral collaboration	Lack of training, recognition	Migration of residents
Ismail et al. (11)	FF	N/A	N/A	B	FF	B	B	B	B	N/A
Suhaili et al. (12)	FF	B	N/A	N/A	N/A	N/A	N/A	N/A	N/A	N/A
Rozhan et al. (13)	N/A	FF	N/A	N/A	N/A	N/A	N/A	N/A	N/A	B
Hod et al. (14)	FF	FF	N/A	N/A	N/A	N/A	N/A	N/A	N/A	N/A
Suraiya et al. (15)	N/A	B	FF	B	N/A	B	B	N/A	B	N/A

KAP = knowledge, attitudes, and practices; FF = facilitating factors; B = barriers; N/A = not available
